# Enhancing Penetration Ability of Semiconducting Polymer Nanoparticles for Sonodynamic Therapy of Large Solid Tumor

**DOI:** 10.1002/advs.202104125

**Published:** 2022-01-06

**Authors:** Xin Wang, Min Wu, Haoze Li, Jianli Jiang, Sensen Zhou, Weizhi Chen, Chen Xie, Xu Zhen, Xiqun Jiang

**Affiliations:** ^1^ MOE Key Laboratory of High Performance Polymer Materials and Technology Department of Polymer Science & Engineering College of Chemistry & Chemical Engineering Nanjing University Nanjing 210023 P. R. China; ^2^ Key Laboratory for Organic Electronics and Information Displays & Jiangsu Key Laboratory for Biosensors Institute of Advanced Materials (IAM) Jiangsu National Synergetic Innovation Center for Advanced Materials (SICAM) Nanjing University of Posts & Telecommunications Nanjing 210023 P. R. China

**Keywords:** large solid tumor therapy, polymer nanoparticles, sonodynamic therapy, tumor hypoxia, tumor penetration

## Abstract

Sonodynamic therapy (SDT) holds growing promise in deep‐seated or large solid tumor treatment owing to its high tissue penetration depth ability; however, its therapeutic efficacy is often compromised due to the hypopermeable and hypoxic characteristics in the tumor milieu. Herein, a semiconducting polymer nanoparticle (SPNC) that synergistically enhances tumor penetration and alleviates tumor hypoxia is reported for sonodynamic therapy of large solid tumors. SPNC comprises a semiconducting polymer nanoparticle core as a sonodynamic converter coated with a poly (ethylene glycol) corona. An oxygen‐modulating enzyme, catalase, is efficiently conjugated to the surface of nanoparticles via the coupling reaction. Superior to its counterpart SPNCs (SPNC2 (84 nm) and SPNC3 (134 nm)), SPNC with the smallest size (SPNC1 (35 nm)) can efficiently penetrate throughout the tumor interstitium to alleviate whole tumor hypoxia in a large solid tumor model. Upon ultrasound (US) irradiation, SPNC1 can remotely generate sufficient singlet oxygen to eradicate tumor cells at a deep‐tissue depth. Such a single treatment of SPNC1‐medicated sonodynamic therapy effectively inhibits tumor growth in a large solid tumor mouse model. Therefore, this study provides a generalized strategy to synergistically overcome both poor penetration and hypoxia of large tumors for enhanced cancer treatment.

## Graphical abstract

Semiconducting polymer nanoparticles (SPNCs) are developed for sonodynamic therapy of large solid tumors. SPNC with the smallest size (SPNC1) can efficiently penetrate throughout the tumor interstitium to alleviate the whole tumor hypoxia and generate sufficient singlet oxygen to eradicate tumor cells at a deep‐tissue depth upon ultrasound irradiation, leading to the enhanced antitumor efficacy of large solid tumor.

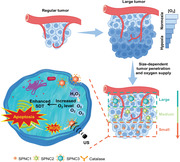

## 1 Introduction

Traditional photodynamic therapy (PDT) that utilizes photoirradiation to eradicate tumors suffers from a relatively low tissue penetration depth (<0.5 cm) of light, restricting PDT to relatively small, superficial tumors.^[^ ^1^ ^]^ Sonodynamic therapy (SDT), a novel noninvasive therapeutic modality for cancer therapy, has high tissue penetration depth (>10 cm) due to the negligible tissue attenuation coefficient of ultrasound (US), permitting the application of SDT to treat deep‐seated or large solid tumors.^[^ ^2^ ^]^ The sonosensitizers are essential for SDT. Similar to the PDT progress, upon US irradiation, the sonosensitizers absorb energy and can be activated from the ground state to the excited state of higher energy. Afterward, they transfer energy to surrounding molecules such as oxygen to generate cytotoxic reactive oxygen species (ROS) such as singlet oxygen (^1^O_2_) to induce cell apoptosis.^[^ ^3^ ^]^ In recent years, various organic molecules including porphyrin derivatives,^[^ ^4^ ^]^ chlorophyll derivatives,^[^ ^5^ ^]^ rose bengal (RB),^[^ ^6^ ^]^ curcumin,^[^ ^7^ ^]^ methylene blue (MB),^[^ ^8^ ^]^ and indocyanine green (ICG)^[^ ^9^ ^]^ which can generate singlet oxygen （^1^O_2_） under light irradiation have been studied as sonosensitizers to generate ^1^O_2_ under US irradiation for SDT.

Despite the high tissue penetration depth of the SDT modality, the hypopermeable and oxygen deficiency characteristics in the tumor microenvironment limit the sonodynamic therapeutic effect in cancer therapy.^[^ ^10^ ^]^ Sonosensitizers inherently require an adequate supply of oxygen to generate sufficient ROS, but the interior hypovascular solid tumors often encounter hypoxia and thus inevitably decrease the therapeutic efficacy of SDT.^[^ ^11^ ^]^ Incorporation of in situ oxygen‐generating materials such as enzymes^[^ ^11a^ ^]^ or oxygen carriers such as hemoglobin^[^ ^11b^ ^]^ and perfluorocarbon^[^ ^12^ ^]^ into sonosensitizers to increase tumor oxygenation is a regulatory strategy to reduce the tolerance of hypoxic tumors to SDT. However, the penetration ability of sonosensitizers in solid tumors has rarely been evaluated. Especially in large solid tumors with hypopermeable characteristics, the penetration ability of sonosensitizers is a vital factor, since the ROS generated by sonosensitizers only induce nearby tumor cell apoptosis.^[^ ^13^ ^]^ Therefore, a new sonodynamic therapeutic system is urgently needed to synergistically enhance tumor penetration and alleviate tumor hypoxia in a large tumor model.

As an emerging category of organic theranostic agents, semiconducting polymer nanoparticles (SPNs) transformed from highly *π*‐electron delocalized semiconducting polymers have high biocompatibility, high chemical flexibility, and tunable sizes.^[^ ^14^ ^]^ To date, SPNs have been explored for a series of biomedical applications including optical imaging,^[^ ^15^ ^]^ phototherapy,^[^ ^16^ ^]^ and biological regulation.^[^ ^17^ ^]^ However, the sonodynamic therapeutic effect of SPNs as sonosensitizers for cancer therapy has yet to be exploited.

**Figure 1:**
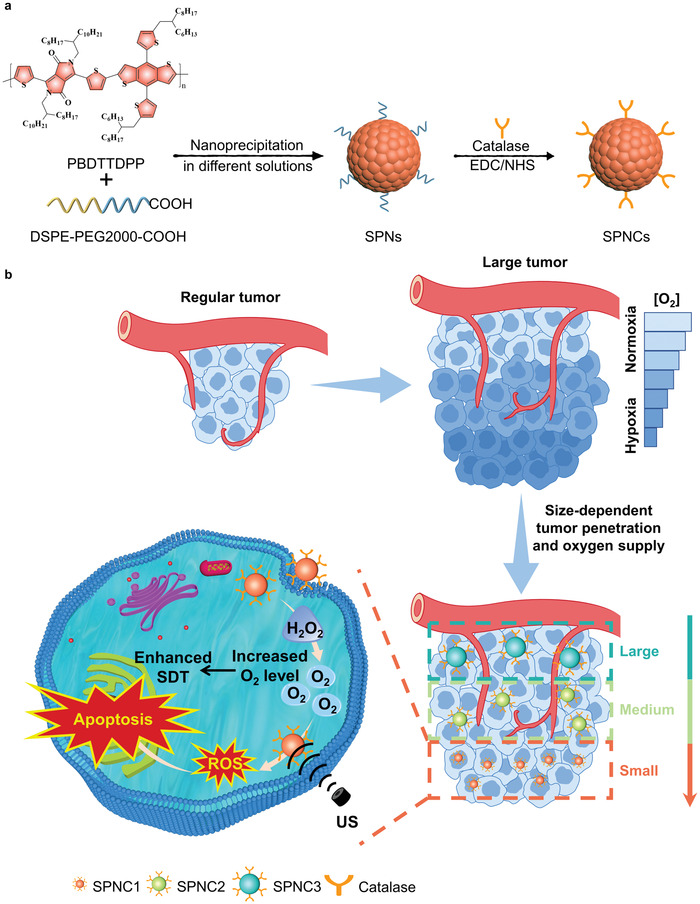
a) Chemical structure of PBDTTDPP and schematic illustration of the preparation of SPNCs of different sizes (SPNC1, SPNC2, and SPNC3). b) Schematic illustration of size‐dependent penetration ability to increase tumor oxygenation for enhanced sonodynamic therapy of SPNC1 in the large tumor model.

In this study, we report the synthesis of US excitable semiconducting polymer nanoparticles (termed as SPNC) that can synergistically overcome both poor penetration and hypoxia in large solid tumors for enhanced SDT. SPNC is composed of a semiconducting polymer nanoparticle core as a sonodynamic converter, conjugated with an oxygen‐modulating enzyme, catalase, via a coupling reaction between the amino group of catalase and the carboxyl group of SPN (**Figure** **1**a). Current studies have demonstrated that nanoparticles with sizes below 60 nm show substantially enhanced penetrating behavior in tumor tissues.^[^ ^18^ ^]^ To verify the vital role of particle size in controlling the penetration of sonosensitizers in large solid tumors, three SPNCs of different sizes are fabricated. Catalase is chosen to react with hydrogen peroxide overproduced in the tumor microenvironment (TME) to generate oxygen in situ to improve tumor hypoxia for enhanced SDT. After systemic administration, all these SPNCs can accumulate in the tumor site through passive targeting. Only SPNC with the smallest size (SPNC1) can preferentially penetrate inside the solid tumor of living mice, thus increasing oxygenation in the inner solid tumor, and thereby boosting the sonotherapeutic effect of large solid tumors upon US irradiation (Figure 1b). As such, SPNC1‐mediated sonodynamic therapy synergistically conquers both poor penetration and hypoxia, contributing to effective large solid tumor inhibition in the mouse model.

## 2 Results and Discussion

### 2.1 Synthesis and In Vitro Characterization

To make SPNC responsive to deep‐tissue penetrating US, a new sonodynamic semiconducting polymer (SP), PBDTTDPP, was designed and synthesized via palladium‐catalyzed Stille polymerization of (4,8‐Bis(5‐(2‐hexyldecyl)thiophen‐2‐yl)benzo[1,2‐b:4,5‐b′]dithiophene‐2,6‐diyl)bis(trimethylstannane) (BDTT) and 1,4‐bis(5‐bromothiophen‐2‐yl)‐2,5‐bis(2‐otcyldodecylpyrrolo[3,4‐c]pyrrole‐3,6‐dione (DPP) (Figure S1, Supporting Information). Proton nuclear magnetic resonance (^1^H NMR) spectroscopy and Fourier transform infrared (FTIR) spectroscopy confirmed the successful synthesis of PBDTTDPP (Figures S2 and S3, Supporting Information). The molecular weight of SP was determined to be ≈25 914 Da by gel permeation chromatography (Figure S4, Supporting Information). Nanoprecipitation was used to transform SP into water‐soluble SPNs of different sizes (termed SPN1, SPN2, and SPN3) in the presence of an amphiphilic polymer, 1,2‐distearoyl‐sn‐glycero‐3‐phosphoethanolamine‐*N*‐[carboxy(polyethylene glycol)‐2000] (DSPE−PEG2000‐COOH). The obtained SPNs were further covalently linked with catalase, affording SPNCs (termed SPNC1, SPNC2, and SPNC3).

**Figure 2:**
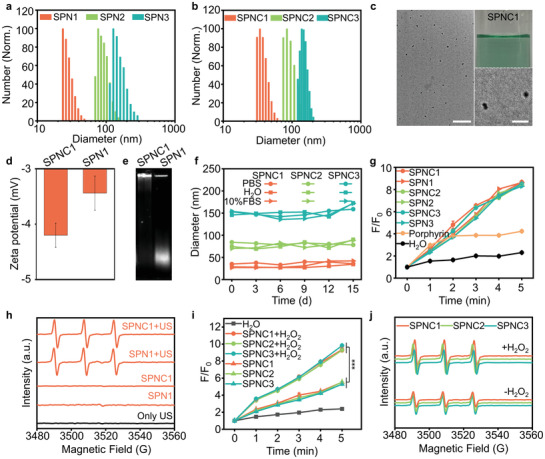
In vitro characterization of SPNs and SPNCs. a) DLS profiles of SPN1, SPN2, and SPN3. b) DLS profiles of SPNC1, SPNC2, and SPNC3. c) Nanoparticle solutions (25 µg mL^–1^) and TEM images of SPNC1. Scale bar (left) = 200 nm. Scale bar (right) = 50 nm. d) Zeta potential of SPN1 and SPNC1. e) Agarose gel electrophoresis of SPNC1 and SPN1. f) Hydrodynamic diameter stability of SPNC1, SPNC2, and SPNC3 after different days in 1× phosphate buffer solution (PBS), H_2_O, and PBS containing 10% serum. g) ^1^O_2_ generation of porphyrin, SPNs, and SPNCs after different times of US irradiation (50 kHz, 1 W cm^–2^, 50% duty cycle) measured by fluorescence intensity enhancement (F/F_0_) of SOSG. h) ^1^O_2_ generation of SPN1 and SPNC1 with or without US irradiation (50 kHz, 1 W cm^–2^, 50% duty cycle) measured by ESR spectra. i) ^1^O_2_ generation of SPNCs measured by fluorescence intensity enhancement (F/F_0_) of SOSG under the hypoxic environment in the presence or absence of H_2_O_2_ (50 × 10^−6^ m). j) ^1^O_2_ generation of SPNCs measured by ESR spectra under hypoxia environment in the presence or absence of H_2_O_2_ (50 × 10^−6^ m). Error bars represent the standard deviations of three separate measurements (*n* = 3). ***p < 0.001.

The physical properties of SPNs and SPNCs were first studied. Dynamic light scattering (DLS) showed that the average hydrodynamic diameters of the SPNs were ≈26 nm (SPN1), 76 nm (SPN2), and 123 nm (SPN3) with polydispersity indices (PDIs) of 0.146, 0.173, and 0.195 respectively (**Figure** **2**a). After the bioconjugation reaction, the hydrodynamic diameters of SPNCs increased to ≈35 nm (SPNC1), 84 nm (SPNC2), and 134 nm (SPNC3) with PDIs of 0.123, 0.177, and 0.207, respectively (Figure 2b). The obtained nanoparticle solutions were translucent and transmission electron microscopy (TEM) images revealed that the obtained SPNs and SPNCs had a spherical morphology (Figure 2c, and Figures S5 and S6, Supporting Information). The larger sizes measured by DLS relative to those measured by TEM are due to the shrinkage of SPNCs in the dry state of TEM. After conjugation of catalase, SPNCs showed a more negative surface charge relative to the counterpart SPNs (Figure 2d and Figure S7, Supporting Information). The migration distance of SPNCs in agarose gel electrophoresis was also much lower than that of their counterpart SPNs (Figure 2e and Figure S8, Supporting Information). These results implied the successful conjugation of catalase with SPNs. The weight ratio of catalase to the SPNC core was calculated to be 68.7%, 66.1%, 64.6%, as determined by the Bradford protein assay. Additionally, no obvious change in hydrodynamic diameters of the SPNCs during storage in 1× phosphate buffer solution (PBS), H_2_O, and PBS containing 10% serum for at least 15 days indicated their excellent colloidal stability (Figure 2f).

The sonodynamic properties of SPNs and SPNCs were then evaluated using the fluorescence indicator singlet oxygen sensor green (SOSG) and electron spin resonance (ESR). 2,2,6,6‐Tetramethylpiperide was used as the singlet oxygen trapping agent to mix with the respective SPNs and SPNCs. Similar to laser irradiation, upon US irradiation, the SOSG fluorescence intensity at 520 nm of both the SPN and SPNC groups was similarly enhanced by ≈8.6‐fold after 5 min irradiation, which was higher than that of commercial porphyrin materials (Figure 2g and Figure S9, Supporting Information). Significant ESR signals induced by singlet oxygen could also be observed in both the SPN and SPNC groups upon US irradiation (Figure 2h and Figure S10, Supporting Information). Additionally, the conformation of SPNCs remained unchanged after US irradiation (Figure S11, Supporting Information). These data clearly proved the outstanding sonodynamic effect of SPNs and SPNCs. Moreover, catalase and the size of SPNCs showed minimal influence on the sonodynamic properties of SPNCs.

To further investigate catalase‐regulated sonodynamic behaviors in the hypoxic tumor microenvironment, the generation of ^1^O_2_ by SPNCs under US irradiation and hypoxic conditions was monitored by SOSG and ESR. In the absence of H_2_O_2_, the SOSG fluorescence intensity in the SPNC group was enhanced only by ≈5.4‐fold upon US irradiation, indicating the weakened sonodynamic behaviors of SPNCs under hypoxic conditions (Figure 2i). In contrast, in the presence of H_2_O_2_, the SOSG fluorescence intensity dramatically increased to ≈9.5‐fold upon US irradiation of SPNCs due to oxygen generation through the catalytic reaction between catalase and H_2_O_2_ (Figure 2i). Similar ^1^O_2_ generation efficiency of SPNCs with and without the addition of H_2_O_2_ was further observed using ESR (Figure 2j). Thus, these data verified that the presence of catalase in SPNCs can effectively elevate the generation of ^1^O_2_ under hypoxia in the presence of H_2_O_2_ and that the ^1^O_2_ generation efficiency was also irrelevant to the size of SPNCs.

### 2.2 In Vitro Studies of Sonodynamic Therapy

**Figure 3:**
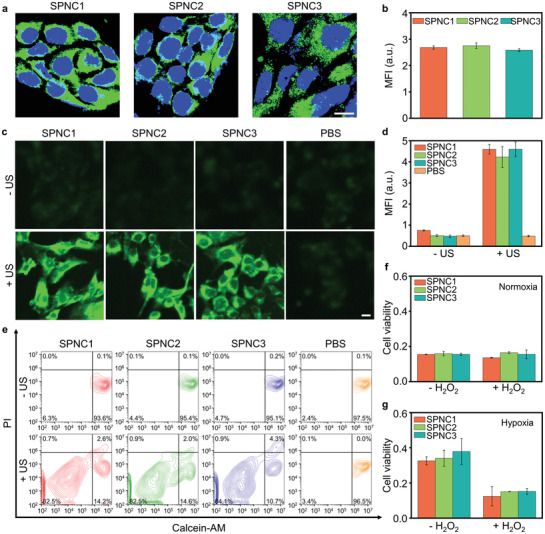
In vitro studies of sonodynamic therapy. a) Confocal fluorescence images and b) mean fluorescence intensity (MFI) of 4T1 cancer cells after incubation with SPNC1, SPNC2, and SPNC3 (25 µg mL^–1^) for 4 h. Blue fluorescence indicated the cell nucleus, and the green fluorescence indicated the signal from BODIPY doped SPNCs. Scale bar = 10 µm. c) Confocal fluorescence images and d) MFI of 4T1 cancer cells after incubation with SPNC1, SPNC2, and SPNC3 (25 µg mL^–1^) for 4 h, followed by staining with DCFH‐DA with or without US irradiation (50 kHz, 1 W cm^–2^, 50% duty cycle) for 1 min. Scale bar = 10 µm. e) Flow cytometry quantification assay of 4T1 cancer cells incubated with SPNC1, SPNC2, and SPNC3 (25 µg mL^–1^) for 4 h with or without US irradiation (50 kHz, 1 W cm^–2^, 50% duty cycle) for 1 min. The cells were stained with calcein‐AM and propidium iodide (PI). Relative cell viability of 4T1 cancer cells after incubation with SPNC1, SPNC2, and SPNC3 (25 µg mL^–1^) with US irradiation (50 kHz, 1 W cm^–2^, 50% duty cycle) under f) normoxic or g) hypoxic environment in the presence or absence of H_2_O_2_(50 × 10^−6^ m). Error bars represent the standard deviations of three separate measurements (*n* = 3).

The cellular uptake of SPNCs was studied using 4T1 cancer cells or RAW 264.7 macrophages. A fluorescent dye (boron‐dipyrromethene (BODIPY)) was doped into nanoparticles (4 w/w%) to endow nanoparticles with fluorescent properties. After treatment with BODIPY‐doped SPNCs for 4 h, 4T1 cancer cells or RAW 264.7 macrophages were imaged by confocal fluorescence microscopy. The relative mean fluorescence intensities (MFIs) of SPNC‐treated cells were almost identical, demonstrating the similar endocytosis of SPNCs in 4T1 cancer cells or RAW 264.7 macrophages probably due to the similar PEG segments and surface zeta potentials for SPNCs (**Figure** **3**a,b, Figure S12, Supporting Information). Next, the in vitro sonodynamic therapeutic efficacy of SPNCs was studied. 2’,7’‐Dichlorodihydrofluorescein diacetate (DCFH‐DA) was used as the ^1^O_2_ fluorescent turn‐on indicator to detect intracellular ^1^O_2_ generation. After US irradiation, remarkable enhancement in the fluorescence intensity of the SPNC‐treated cells was observed compared with the groups without US irradiation (Figure 3c,d). To further examine the ^1^O_2_‐induced cytotoxicity of SPNCs, 4T1 cells were stained with calcein‐AM and propidium iodide (PI) to detect viable and dead cells through immunofluorescence staining and flow analysis, respectively. After treatment with SPNCs, the population of dead cells was more than 80% under US irradiation, while negligible dead cells were found in the PBS group or in the absence of US irradiation (Figure 3e and Figure S13, Supporting Information). Afterward, a methyl thiazolyl tetrazolium (MTT) assay was applied for further quantitative cell viability tests under normoxic or hypoxic conditions. After incubation with different concentrations of SPNCs for 24 h, the cytotoxicity of 4T1 cells was nearly negligible (Figure S14, Supporting Information). Under normoxic conditions with US irradiation, the cell viabilities of SPNC‐treated cells decreased to below 20% in the presence or absence of H_2_O_2_ (Figure 3f), indicating the promising SDT effect of SPNCs under normoxic conditions. However, under hypoxic conditions with US irradiation, the cell viability of SPNC‐treated 4T1 cells only reached 35% in the absence of H_2_O_2_ (Figure 3g), indicating the weakened SDT therapeutic effect of SPNCs under hypoxic conditions. In contrast, in the presence of H_2_O_2_, the hypoxic conditions could be alleviated due to the production of oxygen in cells through the catalytic reaction between catalase and H_2_O_2_, and thus the cell viability of SPNC‐treated 4T1 cells could further decrease to below 20% with US irradiation (Figure 3g and Figure S15, Supporting Information). These results verified that SPNCs could generate sufficient ^1^O_2_ to overcome the challenge of SDT under tumor hypoxic conditions.

### 2.3 In Vivo Sonodynamic Therapy

**Figure 4:**
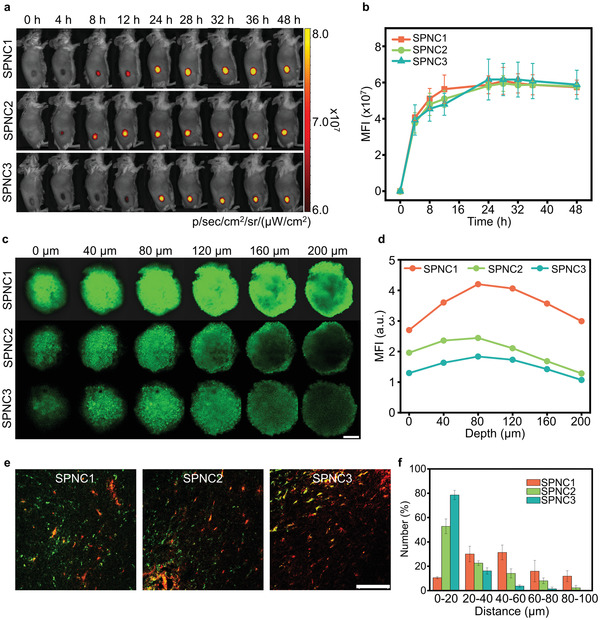
In vivo fluorescence imaging and size‐dependent tumor penetration ability of SPNCs. a) Fluorescence images and b) MFI of tumor sites of 4T1 tumor‐bearing living mice at different time points after intravenous injection of NCBS‐doped SPNC1, SPNC2, and SPNC3 (0.2 mL, 200 µg mL^–1^). c) Z‐stack CLSM images of 4T1 3D MCTs incubated with BODIPY‐doped SPNC1, SPNC2, and SPNC3 for 12 h (25 µg mL^–1^). Scale bar = 200 µm. d) Fluorescence intensity of 4T1 3D MCTs at different depths. e) In vivo penetration behaviors of SPNC1, SPNC2, and SPNC3 in tumor sites of 4T1 tumor‐bearing living mice after 24 h intravenous injection of nanoparticles. Green fluorescence indicated the location of nanoparticles and red fluorescence indicated the location of blood vessels. Scale bar = 100 µm. f) Semi‐quantitative analysis of the distance between nanoparticles and tumor blood vessels after 24 h intravenous injection of nanoparticles. Error bars represent the standard deviations of three separate measurements (*n* = 3).

To identify the biodistribution and the optimal in vivo sonodynamic therapeutic timepoint for these nanoparticles, all the nanoparticles were doped with 4 w/w% near‐infrared (NIR) fluorescent dye (silicon 2,3‐naphthalocyanine bis(trihexylsilyloxide) (NCBS)) to obtain NIR fluorescent derivatives. NIR fluorescence imaging was conducted in the subcutaneous 4T1 tumor mouse model. After intravenous injection of NCBS‐doped SPNC1, SPNC2, or SPNC3, NIR fluorescence images were longitudinally acquired (**Figure** **4**a). For these nanoparticle‐treated mice, the fluorescence of tumors gradually increased and reached a similar maximum MFI at 24 h postinjection time (Figure 4b). In addition, an ex vivo biodistribution study of SPNCs at 24 h postinjection showed that all these nanoparticles displayed similar tissue biodistribution with the maximum accumulation in the tumor, followed by the liver, spleen, and other organs (Figure S16, Supporting Information). Such similar tissue biodistribution in the tumor and major organs of these nanoparticles is probable due to their analogous hydrophilic PEG segment and similar negative surface charge.

To evaluate the penetrating behaviors of these nanoparticles, 4T1 three‐dimensional multicellular tumor spheroids (MCTS) were established to mimic solid tumors and the penetration depth of these nanoparticles was measured. The fluorescence of SPNC1 was detected at obviously greater depth than that of SPNC2 and SPNC3 (Figure 4c). At a depth of 200 µm, the fluorescence intensity of SPNC1 was 2.3‐fold and 2.8‐fold higher than that of SPNC2 and SPNC3, respectively (Figure 4d). These results indicated the size‐dependent penetration behavior of these nanoparticles in the MCTS models. The penetrating behaviors of these nanoparticles in the xenografted 4T1 tumors were then assessed via immunofluorescence staining followed by quantitative analysis (Figure 4e,f). The red fluorescence signals from the Alexa‐594‐conjugated CD31 antibody indicated the location of tumor blood vessels. The green fluorescence signals were from the BODIPY‐doped SPNCs. After intravenous injection of these nanoparticles for 24 h, more than 50% of SPNC2 or SPNC3 only accumulated around tumor blood vessels, and no obvious fluorescence signals were detected inside the tumor interstitium. In comparison, 80% of SPNC1 penetrated across the blood vessels and dispersed throughout the tumor interstitium. This verified that SPNC with the smallest size (SPNC1) showed the best penetration ability in the tumor.

**Figure 5:**
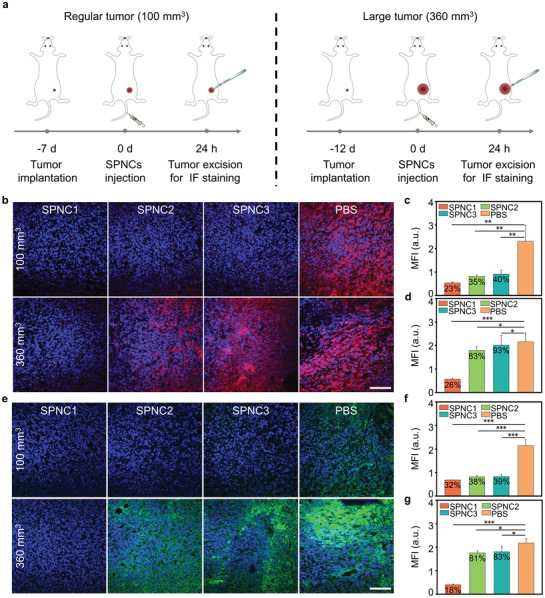
a) Schematic illustration of the timeline for tumor model implantation with different tumor sizes and SPNCs‐mediated tumor oxygenation in the regular and large tumor models. b) Immunofluorescence staining and c,d) MFI of HIF‐1*α* in the regular tumor model (100 mm^3^) and large tumor model (360 mm^3^). Blue fluorescence indicated the cell nucleus, and the red fluorescence indicated the signal from anti‐HIF‐1*α* antibody. Scale bar = 100 µm. e) Immunofluorescence staining and f,g) MFI of pimonidazole in the regular tumor model (100 mm^3^) and the large tumor model (360 mm^3^). Blue fluorescence indicated the cell nucleus, and the green fluorescence indicated the signal from anti‐pimonidazole. Scale bar = 100 µm. Error bars represent the standard deviations of three separate measurements (*n* = 3). *p < 0.05, **p < 0.01 and ***p < 0.001.

To verify the ability of these nanoparticles to mitigate tumor hypoxia, an endogenous hypoxia biomarker hypoxia‐inducible factor‐1*α* (HIF‐1*α*) antibody and the hypoxyprobe pimonidazole were used to label hypoxic regions via immunofluorescence staining. Two different sized 4T1 tumor models (regular tumor model, 100 mm^3^; large tumor model, 360 mm^3^) were established to further examine the impact of penetrating behaviors of these nanoparticles on tumor hypoxia because regular tumors are relatively well‐vascularized but the interior of large tumors are usually hypovascular (**Figure** **5**a).^[^ ^19^ ^]^ For the regular tumor model, the SPNC1‐, SPNC2‐, and SPNC3‐treated groups showed tremendously decreased red fluorescence, which was 23%, 35%, and 40% relative to that of the PBS‐treated group, respectively, indicating a significantly reduced expression level of HIF‐1*α* (Figure 5b,c). This result was mainly attributed to the catalase activity in these nanoparticles. However, in the large tumor model, only the red fluorescence intensity of the SPNC1‐treated group relative to that of the PBS‐treated group decreased to 26%, which was 3.2‐ and 3.6‐fold lower than SPNC2 (83%) and SPNC3 (93%), respectively (Figure 5d). This result indicated that tumor oxygenation was also dependent on the penetration ability of nanoparticles due to the hypovascular character in the large tumor model. Similar tendencies were observed through immunofluorescence staining with the hypoxyprobe pimonidazole (Figure 5e–g). These data suggested that only the SPNC1‐treated group effectively alleviated tumor hypoxia in the large tumor model, indicating that both the in situ oxygen generation ability and penetration ability of SPNC_S_ were vital to mitigate tumor hypoxia in the large tumor model.

**Figure 6:**
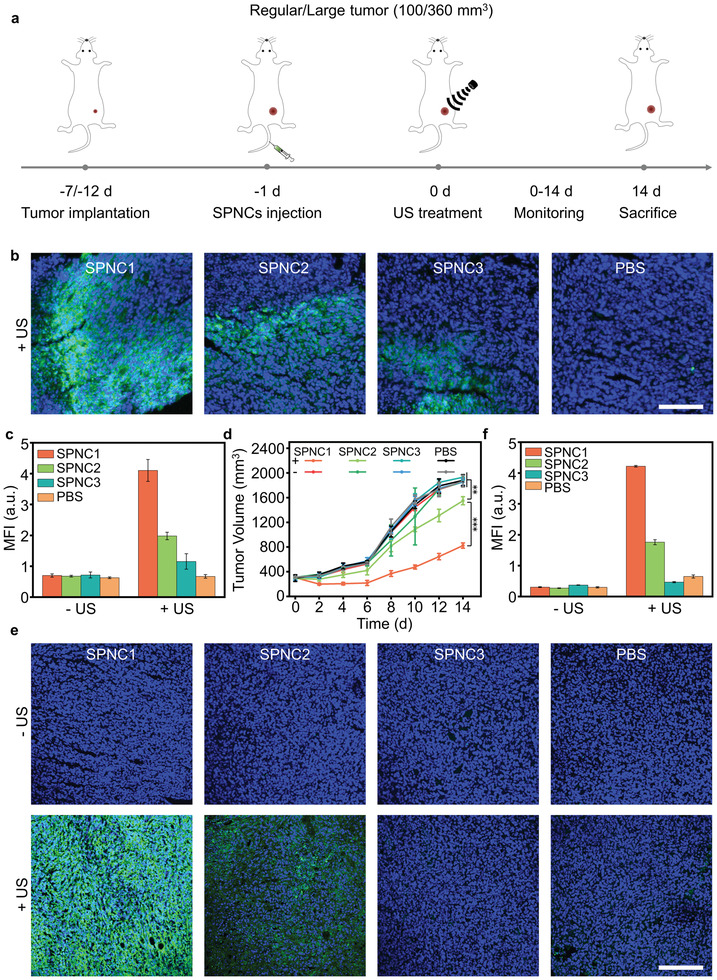
a) Schematic illustration of the timeline for tumor model implantation with different tumor sizes and SPNCs‐mediated sonodynamic therapy in the regular and large tumor models. b) Confocal fluorescence images and c) MFI of tumors from the large 4T1 tumor‐bearing mice after intravenous injection of SPNC1, SPNC2, SPNC3, and PBS treated with SOSG in the presence of US irradiation. Blue fluorescence indicated the cell nucleus, and the green fluorescence indicated the signal from SOSG. Scale bar = 100 µm. d) Tumor growth curves of different groups of mice in the large tumor model after systemic treatment with PBS, SPNC1, SPNC2, or SPNC3 in the presence or absence of US irradiation. e) Immunofluorescence staining and f) MFI of caspase‐3 of tumors from large 4T1 tumor‐bearing mice after intravenous injection of SPNC1, SPNC2, SPNC3, and PBS in the presence or absence of US irradiation. Blue fluorescence indicated the cell nucleus, and the green fluorescence indicated the signal from anti caspase‐3 antibody. Scale bar = 100 µm. Error bars represent the standard deviations of three separate measurements (*n* = 5). **p < 0.01 and ***p < 0.001.

The in vivo sonodynamic therapeutic efficiency of these nanoparticles was further examined in 4T1 tumor‐bearing mice (**Figure** **6**a). US irradiation of the tumor was conducted at the maximum accumulation timepoint (24 h post‐intravenous administration of these nanoparticles). The generation of ^1^O_2_ was measured by detecting the fluorescence signals of intratumorally injected SOSG. For both regular and large tumor models, there were obvious green fluorescence signals of SOSG and a significant increase in the MFIs of the tumors of SPNC1, SPNC2, and SPNC3‐treated mice, respectively, after US irradiation compared to that of the groups without US irradiation (Figure 6b and Figure S17, Supporting Information), confirming the ^1^O_2_ generation of these nanoparticles in the tumor region. Not surprisingly, for the large tumor model, upon US irradiation, SPNC1‐treated mice exhibited the most significant increase in the fluorescence signals of SOSG, showing a 1.3‐ or 1.9‐fold increase compared with SPNC2‐treated or SPNC3‐treated mice (Figure 6c).

The in vivo cancer therapeutic efficacy was evaluated by monitoring the growth of tumors every two days for two weeks. After therapy, the regular tumors in SPNC1‐, SPNC2‐, and SPNC3‐treated mice were completely inhibited after US irradiation, whereas the mice treated with nanoparticles without US irradiation showed similar tumor growth relative to PBS‐treated mice (Figure S18, Supporting Information). For the large tumor model, after US irradiation, SPNC3 failed to inhibit tumor growth, and the tumor growth of SPNC2‐treated mice was only slowed; however, the tumor control rate of SPNC1‐treated mice reached 66.6%, which was 3.5‐fold higher than that in SPNC2‐treated mice (Figure 6d and Figure S19, Supporting Information). The therapeutic efficacy was further confirmed by immunofluorescence caspase‐3 staining (Figure 6e and Figure S20, Supporting Information). For the large tumor model, no obvious apoptosis was observed for the tumors of PBS or nanoparticle‐treated mice without US irradiation, while stronger green fluorescence from apoptotic cells was observed for the tumors of SPNC1‐treated mice with US irradiation, which was 2.5‐ or 9.0‐fold higher relative to that of SPNC2‐treated and SPNC3‐treated mice with US irradiation, respectively (Figure 6f). These data indicated that the sole improved tumor oxygenation strategy only enhanced the sonodynamic therapeutic efficacy in the regular tumor model but failed to inhibit tumor growth in the large tumor model. This result was consistent with the previous literature that sonosensitizers with in situ oxygen supply ability showed effective tumor growth inhibition in regular tumor models (initial tumor size: 80–120 mm^3^) (Table S1, Supporting Information).^[^ ^4^, ^10^, ^11^, ^12^, ^20^ ^]^ However, for the large tumor model, only SPNC1 could penetrate throughout the tumor interstitium to alleviate whole tumor hypoxia and eradicate tumor cells at a deep‐tissue depth, leading to effective SDT for enhanced antitumor efficacy.

The biosafety of the proposed SDT was further verified by monitoring the changes in body weights and histological analysis of the major organs. No noticeable weight loss was observed for mice after all these treatments over the 14 days (Figure S21, Supporting Information). No obvious histological or pathological changes were observed in the hematoxylin and eosin (H&E) staining of major organs such as hearts, livers, spleens, lungs, and kidneys from mice after different treatments (Figure S22, Supporting Information). Furthermore, the serum levels of liver function indicators including alanine transaminase (ALT), aspartate aminotransferase (AST), and alkaline phosphatase (ALP) (Figure S23, Supporting Information) and kidney function indicators including blood urea nitrogen (BUN) and creatinine (CREA) (Figure S24, Supporting Information) were in the normal levels in healthy mice after injection of SPNCs for different times (1, 7, and 14 days). The in vivo fate of SPNCs was monitored through long‐term NIR fluorescence recording of SPNC1 after injection into healthy mice. After injection, the NIR fluorescence signals of SPNC1 first accumulated in the liver region and transferred to the intestinal region over time. Afterward, the NIR fluorescence signals from the intestine region continuously decreased to the background level at 4 days postinjection, demonstrating the fast clearance of SPNC1 via hepatobiliary excretion in living mice (Figure S25, Supporting Information). These results demonstrated the good in vivo biosafety of these nanoparticles for cancer therapy.

## 3 Conclusion

In summary, we have developed a semiconducting polymer nanoparticle (SPNC1) that can synergistically enhance tumor penetration and in situ supply O_2_ for enhanced sonodynamic therapy of a large tumor model. In vitro results validated that SPNC1 showed outstanding ^1^O_2_ generation efficiency upon US irradiation. Owing to the catalase and smallest size, SPNC1 could not only penetrate throughout the tumor interstitium but also react with H_2_O_2_ overproduced in the TME to generate O_2_. Therefore, after intravenous administration, the whole tumor hypoxia in SPNC1‐treated mice was remarkably alleviated in the large tumor model compared with that in other groups. As a result, SPNC1‐mediated sonodynamic therapy enabled effective therapeutic efficacy against large tumor models, which could hardly be achieved by the counterpart nanoparticles (SPNC2 and SPNC3). This result revealed that the penetration ability of sonosensitizers played a vital factor in the sonodynamic therapy of large tumors since the sole in situ oxygen supply strategy for SDT was only achieved in the regular tumor model. SPNC1 represents a unique type of sonodynamic nanoagent that can synergistically enhance tumor penetration ability and in situ increase tumor oxygenation in a large tumor model. Thus, our study provides a generalized strategy to exert synergetic action to overcome both poor penetration and hypoxia of large tumors for enhanced sonodynamic therapy.

## Conflict of Interest

The authors declare no conflict of interest.

## Supporting information

Supporting Information

## Data Availability

The data that support the findings of this study are available from the corresponding author upon reasonable request.
