# Supplementary material for: Enhancing Penetration Ability of Semiconducting Polymer Nanoparticles for Sonodynamic Therapy of Large Solid Tumor
Source: Adv Sci (Weinh). 2022 Jan 6;9(6):2104125. doi: 10.1002/advs.202104125 (PMC8867194; doi:10.1002/advs.202104125)
Supplement: Supplementary file 1 — Supporting Information [file ADVS-9-2104125-s001.pdf]

© 2020 Wiley-VCH GmbH

ADVANCED SCIENCE
Open Access

Supporting Information

for *Adv. Sci.*, DOI: 10.1002/advs.202104125

Enhancing penetration ability of semiconducting polymer nanoparticles for sonodynamic therapy of large solid tumor

Xin Wang, Min Wu, Haoze Li, Jianli Jiang, Sensen Zhou, Weizhi Chen, Chen Xie, Xu Zhen*, and Xiqun Jiang**

Supporting Information

Enhancing penetration ability of semiconducting polymer nanoparticles for sonodynamic therapy of large solid tumor

Xin Wang[+], Min Wu[+], Haoze Li, Jianli Jiang, Sensen Zhou, Weizhi Chen, Chen Xie[*], Xu Zhen[*], and Xiqun Jiang[*]

X. Wang, M. Wu, H. Li, J. Jiang, S. Zhou, Dr. W. Chen, Prof. X. Zhen, Prof. X. Jiang

MOE Key Laboratory of High Performance Polymer Materials and Technology, and

Department of Polymer Science & Engineering, College of Chemistry & Chemical

Engineering, Nanjing University, Nanjing, 210023, P. R. China.

E-mail: Xu Zhen: zhenxu@nju.edu.cn and Xiqun Jiang: jiangx@nju.edu.cn

Prof. C. Xie

Key Laboratory for Organic Electronics and Information Displays & Jiangsu Key Laboratory for Biosensors, Institute of Advanced Materials (IAM), Jiangsu National Synergetic Innovation Center for Advanced Materials (SICAM), Nanjing University of Posts & Telecommunications, Nanjing 210023, P. R. China.

E-mail: iamcxie@njupt.edu.cn

[+] These authors contributed equally to this work.

Keywords: sonodynamic therapy, large solid tumor therapy, polymer nanoparticles, tumor penetration, tumor hypoxia

Table of Contents

1. **Experimental section**
2. **Supporting Figures and Tables**
3. **References**

1. Experimental section

Chemicals: All chemicals were purchased from Sigma-Aldrich unless otherwise stated. General chemicals were of the best grade available and were used without further purification. (4,8-Bis(5-(2-hexyldecyl)thiophen-2-yl)benzo[1,2-b:4,5-b']dithiophene-2,6-diyl)bis(trimethylstannane) and 1,4-bis(5-bromothiophen-2-yl)-2,5-bis(2-otcyldodecyl)pyrrolo[3,4-c]pyrrole-3,6-dione were purchased from Nanjing Zhiyan Technology Co., LTD. DSPE-PEG2000-COOH was purchased from Shanghai Proglo Biotechnology Co., LTD. singlet oxygen sensor green was purchased from Molecular Probes. Bradford Protein Quantification Kit was purchased from Nanjing Norvizan Biological Technology Co., LTD. H_2DCFDA was purchased from Dalian Meilun Biotechnology Co., LTD. Methyl thiazolyl tetrazolium (MTT) was purchased from Alfa Aesar. All antibodies were purchased from Abcam.

Characterizations: Proton nuclear magnetic resonance (^{1}H NMR) spectra were recorded by using a BRUKER AVANCE III 400 MHz NMR. GPC data were obtained on Agilent 1100. FTIR spectrum was recorded using a NICOLET iS10. Dynamic light scattering (DLS) was performed on the Brookhaven 90Plus. Transmission electron microscopy (TEM) images were obtained on a JEM-2100 transmission electron microscope. Zeta potentials were obtained on the Malvern Nano-ZS Particle Size. Ultraviolet-Vis spectra were recorded on a Shimadzu UV-3600 spectrophotometer. Fluorescence measurements were carried on a HORIBA FM-4NIR spectrofluorometer. ESR spectra were obtained on the Bruker BioSpin EMX PLUS(PPMS). Fluorescence confocal images were conducted using confocal laser scanning microscopy (Zeiss LSM 710) imaging system. Flow cytometry was conducted on the Aria SORP. Fluorescence images of living mice were acquired with the IVIS Spectrum imaging system (PerkinElmer, Inc.). The ultrasound generator purchased from Hainertec (Suzhou) Co., Ltd. was used for SDT experiments.

Synthesis of PBDTTDPP: (4,8-Bis(5-(2-hecyloctyl)thiophen-2-yl)benzo[1,2-b:4,5-b']dithiophene-2,6-diyl)bis(trimethylstannane) (BDTT) (31.5 mg, 2.79×10^{-2} mmol), 1,4-bis(5-bromothiophen-2-yl)-2,5-bis(2-otcyldecyl)pyrrolo[3,4-c]pyrrole-3,6-dione (DPP) (28.4 mg, 2.79×10^{-2} mmol), $PdCl_2(PPh_3)_2$ (2.4 mg, 3.49×10^{-3} mmol) and 2,6-Di-tert-butylphenol (0.4 mg, 1.99×10^{-3} mmol) were dissolved in 2 mL methylbenzene at a molar ratio of 56:56:7:4. After being degassed three times with freeze-pump-thaw circles, the mixture was reacted at 105 °C under nitrogen atmosphere for 6.5 h. The obtained mixture was precipitated in methanol. The precipitates was washed by methanol for three times and dried under vacuum overnight. ^{1}H NMR (400 MHz, CDCl3, δ): 9.15 (d, 2H), 7.72 (s, 2H), 7.43 (d, 2H), 7.02 (d, 2H), 6.93 (d, 2H), 3.08-2.84 (d, 8H), 1.89 (m, 4H), 1.47-1.02 (m, 112H), 0.87 (d, 24H).

Preparation of SPN1, SPN2, and SPN3: SPNs were prepared by a nanoprecipitation method.

SPN1: A mixture solution containing PBDTTDPP (0.25 mg), DSPE-PEG2000-COOH (5 mg), and THF (1 mL) was prepared. Then the above solution was rapidly injected into the mixture of water (9 mL) and THF (1 mL) under continuous sonication for 2 mins. After sonication, THF was removed with a nitrogen blow. The aqueous solution was filtered through a polyethersulfone (PES) syringe-driven filter (0.22 μm) (Millipore) and washed three times using a 50 K centrifugal filter unit (Millipore) under centrifugation at 3,500 r.p.m. for 15 min.

SPN2: A mixture solution containing PBDTTDPP (0.25 mg), DSPE-PEG2000-COOH (5 mg), and THF (1 mL) was prepared. Then the above solution was injected into the mixture of water (5 mL) and THF (5 mL) under continuous sonication for 2 mins. After sonication, THF was removed with a nitrogen blow. The aqueous solution was filtered through a polyethersulfone (PES) syringe-driven filter (0.22 μm) (Millipore) and washed three times using a 50 K centrifugal filter unit (Millipore) under centrifugation at 3,500 r.p.m. for 15 min.

SPN3: A mixture solution containing PBDTTDPP (0.25 mg), DSPE-PEG2000-COOH (5 mg) and $CHCl_3$ (1 mL) was prepared. Then the above solution was injected into the mixture of

water (9 mL) and $CHCl_3$ (1 mL) under continuous sonication for 2 mins. After sonication, THF was removed with a nitrogen blow. The aqueous solution was filtered through a polyethersulfone (PES) syringe-driven filter (0.22 μm) (Millipore) and washed three times using a 50 K centrifugal filter unit (Millipore) under centrifugation at 3,500 r.p.m. for 15 min.

For cellular uptake assay**,** penetration assay in 3D tumor spheroids, and in vivo tumor penetration, the BODIPY doped SPNs was prepared by mixing PBDTTDPP (0.25 mg), DSPE-PEG2000-COOH (5 mg), BODIPY 505/515 (10 μL, 1 mg mL^{-1} in DMSO), and THF (1 mL). The following procedures were the same as described above.

For in vivo fluorescence imaging, the NCBS doped SPNs were prepared by mixing PBDTTDPP (0.25 mg), DSPE-PEG2000-COOH (5 mg), NCBS (10 μL, 1 mg mL^{-1} in THF), and THF (1 mL). The following procedures were the same as described above.

The obtained SPNs were finally concentrated to different concentrations by ultrafiltration and used for experiments. The concentration of SPNs was quantified through a Shimadzu UV-3600 spectrophotometer according to their absorption coefficients.

Preparation of SPNC1, SPNC2, and SPNC3: The respective SPNs (50 μg mL^{-1}, 10 mL), 1-Ethyl-3-(3'-dimethylaminopropyl)carbodiimide (10 mg) and N-Hydroxysuccinimide (6 mg) were respectively dissolved in 1 x PBS. After 3 hours of stirring at room temperature, catalase (1 mg mL^{-1}, 2.5 mL) in 1 x PBS was added into the above mixture. The mixture solution was further stirred for 12 h at room temperature to obtain SPNCs. The obtained solution was dialyzed by a 1000 KD dialysis bag to remove residual catalase.

Preparation of porphyrin nanoparticles: A mixture solution containing porphyrin (0.25 mg), DSPE-PEG2000-COOH (5 mg), and THF (1 mL) was prepared. Then the above solution was rapidly injected into a mixture of water (9 mL) and THF (1 mL) under continuous sonication for 2 mins. After sonication, THF was removed with a nitrogen blow. The aqueous solution was filtered through a polyethersulfone (PES) syringe-driven filter (0.22 μm) (Millipore) and

washed three times using a 50 K centrifugal filter units (Millipore) under centrifugation at 3,500 r.p.m. for 15 min. The concentration of porphyrin nanoparticles was quantified through a Shimadzu UV-3600 spectrophotometer according to its absorption coefficient.

Agarose gel electrophoresis: Agarose powder was dissolved in 1×Tris/boric acid/ethylenediaminetetraacetic acid (TBE) buffer by microwave heating and solidified. The samples loaded to the gel were prepared by mixing SPNC or SPN solutions (40 μL), 10% sodium dodecyl sulfate (SDS) solution (5 μL), and 10×loading buffer (5 μL). The electrophoresis was performed on a horizontal electrophoresis system at 120V for 60 min. The result was visualized in an IVIS living imaging system. *In Vitro $^1O_2$ Generation Study*: The generation of $^1O_2$ was measured by singlet oxygen sensor green (SOSG) probe. Briefly, 2.5 μL SOSG was added into SPNCs solution (25 μg mL^{-1}, 3mL), followed by US irradiation (50 kHz, 1 W cm^{-2}, 50% duty cycle) for different times. The fluorescence intensity of SOSG at 520 nm was recorded every 1 min. Fluorescence intensity at 520 nm for each sample before US irradiation (F_0) was also measured. Generation of $^1O_2$ was expressed as the enhancement of fluorescence intensity (F_{sample}/F_0) for each sample. To simulate the hypoxic environment, nitrogen is constantly pumped into the solution.

In vitro photodynamic performance study: The generation of $^1O_2$ was measured by SOSG probe. Briefly, 2.5 μL SOSG was added into SPNC1 solution (25 μg mL^{-1}, 3mL), followed by laser irradiation (680 nm, 0.7 W cm^{-2}) for different times. The fluorescence intensity of SOSG at 520 nm was recorded every 1 min. Fluorescence intensity at 520 nm before laser irradiation (F_0) was also measured. Generation of $^1O_2$ was expressed as the enhancement of fluorescence intensity (F_{sample}/F_0).

Bradford protein assay: The weight ratio of catalase to SPNCs core was measured by a Bradford protein quantification kit. SPNCs (25 μg mL^{-1}, 10 μL), 2 x Bradford reagent, and deionized water (90 μL) were mixed. After 5-10 min, the absorbance of the above mixture at

595 nm was recorded. The weight ratio of catalase to SPNCs core could be calculated by the standard curve of BSA.

Cell culture and in vitro cellular uptake assay: 4T1 cells were cultured by RPMI 1640 medium containing 10% FBS, 100 U mL^{-1} penicillin, and 100 μg mL^{-1} streptomycin at 37 °C in a humidified incubator containing 5% CO_2. The 4T1 cells were dispensed into a dish at a concentration of 5×10^4 cells mL^{-1} and incubated for 24 h. Then the cells were treated with the BODIPY doped SPNCs (25 μg mL^{-1}) for 4 h for cellular uptake. The treated cells were stained with DAPI for nuclei staining. The fluorescence images of the cells were performed by a confocal laser scanning microscopy (CLSM) imaging system. Macrophage cells were cultured by DMEM medium containing 10% FBS, 100 U mL^{-1} penicillin, and 100 μg mL^{-1} streptomycin at 37 °C in a humidified incubator containing 5% CO_2. The macrophage cells were dispensed into a dish at a concentration of 5×10^4 cells mL^{-1} and incubated for 24 h. Then the cells were treated with the BODIPY doped SPNCs (25 μg mL^{-1}) for 4 h for cellular uptake. The treated cells were stained with DAPI for nuclei staining. The fluorescence images of the cells were performed by a confocal laser scanning microscopy imaging system.

Detection of ROS in cells: The intracellular ROS was measured using H_2DCFDA 4T1 cells were incubated in the six-well plate together with the coverslips. After incubation for 4 h, the medium was replaced by fresh medium containing SPNCs (25 μg mL^{-1}), and the cells were then incubated for 4 h and irradiated by US (50 kHz, 1 W cm^{-2}, 50% duty cycle) for 1 min. After that, the cells were washed with PBS, fixed with 4% polyformaldehyde solution, and mixed with H_2DCFDA. The cells were further washed with PBS and imaged by a confocal laser scanning microscopy imaging system.

Detection of O_2 production in cells: The intracellular O_2 production was measured using pimonidazole. 4T1 cells were incubated in the six-well plate together with the coverslips. After incubation for 4 h, the medium was replaced by fresh medium containing SPNCs (25 μg

mL^{-1}), H_2O_2 (50μM), and pimonidazole, and the cells were then incubated for 4 h in the hypoxic bag. After that, the cells were washed with PBS, fixed with 4% polyformaldehyde solution, and mixed with anti-pimonidazole. The cells were dyed with DAPI to mark the nuclei. The cells were further washed with PBS and imaged by a confocal laser scanning microscopy imaging system.

In vitro cytotoxicity assay: 4T1 cells were incubated with SPNCs (25 μg mL^{-1}) for 4 h, followed by US irradiation (50 kHz, 1 W cm^{-2}, 50% duty cycle) for 1 min. The treated cells were stained with calcein-AM (AM, live cell) and propidium iodide (PI, dead cell). The cells were imaged by a CLSM imaging system. For flow cytometry analysis, 4T1 cells were incubated with SPNCs (25 μg mL^{-1}) for 4 h, followed by US irradiation (50 kHz, 1 W cm^{-2}, 50% duty cycle) for 1 min. The cells were collected by dissociation via trypsin, centrifugation, and washing for 3 times with PBS. The treated cells were stained with calcein-AM (AM, live cell) and propidium iodide (PI, dead cell). The flow cytometry analysis was then applied.

The cell viability was determined by a standard methyl thiazolyl tetrazolium (MTT) assay. 4T1 cells were seeded in 96-well plates at an intensity of 6×10^4 cells mL^{-1}. After 12 h incubation, the medium was replaced by a fresh medium containing SPNCs at different concentrations (0, 5, 10, 15, 20, 30 μg mL^{-1}) and the cells were incubated for 24 h. Then MTT solution (5 mg mL^{-1}) was added to the medium. After 4 h, the absorbance of MTT at 570 nm was measured.

To evaluate the US toxicity of SPNCs, 4T1 cells were seeded in 96-well plates at an intensity of 6×10^4 cells mL^{-1}. After 12 h incubation, the medium was replaced by fresh medium containing SPNCs (25 μg mL^{-1}). Then the cells were incubated under normoxic (21% O_2) or hypoxic conditions (0% O_2) for another 1 h, followed by US irradiation (50 kHz, 1 W cm^{-2}, 50% duty cycle) for 1 min and further cultured in an incubator for 24 hours. Then MTT

solution (5 mg mL^{-1}) was added to the medium. After 4 h, the absorbance of MTT at 570 nm was measured.

Penetration assay in 3D tumor spheroids: The 3D multicellular tumor spheroids were constructed and cultured according to a previous method.[1] To investigate the penetration depth of these nanoparticles, 5 mL Eppendorf tubes were used to collect about 25 spheroids each. The 3D tumor spheroids were incubated with the BODIPY doped SPNCs (25 µg mL^{-1}) for 12 h and imaged by a CLSM imaging system.

Tumor models: Female BALB/c mice were bought from the Animal Center of Drum-tower Hospital and performed in accord with guidelines provided by the Animal Care Committee at Nanjing University (IACUC-D2103038). To establish 4T1 tumors, 100 µL of 4T1 cells (5×10^6) in PBS suspension were injected into the flank of each mouse.

In vivo tumor fluorescence imaging: 4T1 tumor-bearing mice were intravenously injected with NCBS doped SPNCs (200 µg mL^{-1}, 200 µL). The mice were imaged using an IVIS spectrum imaging system at 0, 4, 8, 12, 24, 28, 32, 36, 48 h post-injection. After 24 h of post-injection, the tumors and major organs (heart, liver, spleen, lung, and kidney) were collected and imaged using an IVIS Spectrum imaging system. The quantification of fluorescence intensities was performed using Living Image software.

In vivo tumor penetration: The BODIPY doped SPNCs (200 µg mL^{-1}, 200 µL) were intravenously injected into 4T1 tumor-bearing mice. After 24 h, the mice were sacrificed and tumors were collected in 4% paraformaldehyde aqueous solution for 4 h. For histological analysis, the tumors were dehydrated with 30% sucrose solution for 12 h and cut into 9 µm sections after being frozen at optimal cutting temperature (O.C.T.). To stain tumor slices, the tumor sections were incubated with antibody CD31 and the secondary antibody (Alexa 594 conjugated donkey anti-rat antibody). The tumor sections were dyed with DAPI to label the nuclei. The slices were imaged by a CLSM imaging system.

In vivo oxygen improvement: SPNCs (200 μg mL^{-1}, 200 μL) were intravenously injected into regular or large 4T1 tumor-bearing mice respectively. After 24 h, the mice were sacrificed and tumors were collected in 4% paraformaldehyde aqueous solution for 4 h. For histological analysis, the tumors were dehydrated with 30% sucrose solution for 12 h and cut into 9 μm sections after being frozen at optimal cutting temperature (O.C.T.). To stain tumor slices, the tumor sections were incubated with antibody HIF-1α. The tumor sections were dyed with DAPI to label the nuclei. The slices were imaged by a CLSM imaging system.
SPNCs (200 μg mL^{-1}, 200 μL) were intravenously injected into regular or large 4T1 tumor-bearing mice respectively. After 24 h, pimonidazole was intraperitoneally injected into 4T1 tumor-bearing mice. 1 h later, the mice were sacrificed and tumors were collected in 4% paraformaldehyde aqueous solution for 4 h. For histological analysis, the tumors were dehydrated with 30% sucrose solution for 12 h and cut into 9 μm sections after being frozen at optimal cutting temperature (O.C.T.). To stain tumor slices, the tumor sections were incubated with anti-pimonidazole. The tumor sections were dyed with DAPI to label the nuclei. The slices were imaged by a CLSM imaging system.

In vivo $^1O_2$ generation in the tumor: SPNCs (200 μg mL^{-1}, 200 μL) were intravenously injected into regular or large 4T1 tumor-bearing mice respectively. After 24 h, 20 μL SOSG was in situ injected into 4T1 tumor-bearing mice and subsequently irradiated by US (50 kHz, 1 W cm^{-2}, 50% duty cycle) for 5 min. After that, the mice were sacrificed and tumors were collected in the 4% paraformaldehyde aqueous solution for 4 h. For histological analysis, the tumors were dehydrated with 30% sucrose solution for 12 h and cut into 9 μm sections after being frozen at optimal cutting temperature (O.C.T.). The tumor sections were dyed with DAPI to mark the nuclei. The slices were imaged by a CLSM imaging system.

In vivo SDT: When the tumor volume reached to about 100 mm^3, regular 4T1 tumor-bearing mice were randomly divided into 8 groups (n = 5 per group): (1) SPNC1 (200 μg mL^{-1}, 200

μL) + US (50 kHz, 1 W cm^{-2}, 50% duty cycle); (2) SPNC1 (200 μg mL^{-1}, 200 μL); (3) SPNC2 (200 μg mL^{-1}, 200 μL) + US (50 kHz, 1 W cm^{-2}, 50% duty cycle); (4) SPNC2 (200 μg mL^{-1}, 200 μL); (5) SPNC3 (200 μg mL^{-1}, 200 μL) + US (50 kHz, 1 W cm^{-2}, 50% duty cycle); (6) SPNC3 (200 μg mL^{-1}, 200 μL); (7) PBS + US (50 kHz, 1 W cm^{-2}, 50% duty cycle); (8) PBS. At 24 h after intravenous injection, the tumors were treated with US irradiation for 5 mins. The tumor volumes and body weights were recorded every two days. The tumor volume (V) was figured out as V = length × width^2/2. On day 14, mice were sacrificed and the tumor weights were measured. Then tumors were collected in 4% paraformaldehyde aqueous solution for 4 h. For histological analysis, the tumors were dehydrated with 30% sucrose solution for 12 h and cut into 9 μm sections after being frozen at optimal cutting temperature (O.C.T.). To stain tumor slices, the tumor sections were incubated with an anti-caspase-3 antibody. The tumor sections were dyed with DAPI to label the nuclei. The slices were imaged by a CLSM imaging system.

When the tumor volume reached about 360 mm^3, large 4T1 tumor-bearing mice were randomly divided into 8 groups (n = 5 per group) and the following experiments were processed the same as described above.

In vivo metabolism: NCBS doped SPNC1 (200 μg mL^{-1}, 200 μL) were intravenously injected into healthy mice. The mice were imaged using an IVIS spectrum imaging system from day 0 to day 14. After 24 h of post-injection, the major organs (heart, liver, spleen, lung, kidney, and intestine) were collected and imaged using an IVIS Spectrum imaging system. The quantification of fluorescence intensities was performed using Living Image software.

Histological studies: After 14 days of treatments, major organs (heart, liver, spleen, lung, and kidney) of 4T1 tumor-bearing mice were collected and fixed with 4% paraformaldehyde for hematoxylin and eosin (H&E) staining.

Biochemical analysis: SPNCs (200 μg mL^{-1}, 200 μL) were intravenously injected into healthy mice. For blood biochemistry detection, mice blood was collected on day 1, day 7, and day 14. The serum was harvested by centrifuging for biochemical analysis.

Statistical analysis: All data were expressed as means ± SD. Statistical differences were performed using student's t-test and one-way ANOVA with Excel. $P < 0.05$ was considered statistically significant. Significant p values are demoted by *$p < 0.05$, **$p < 0.01$ and ***$p < 0.001$.

2. Supporting Figures and Tables

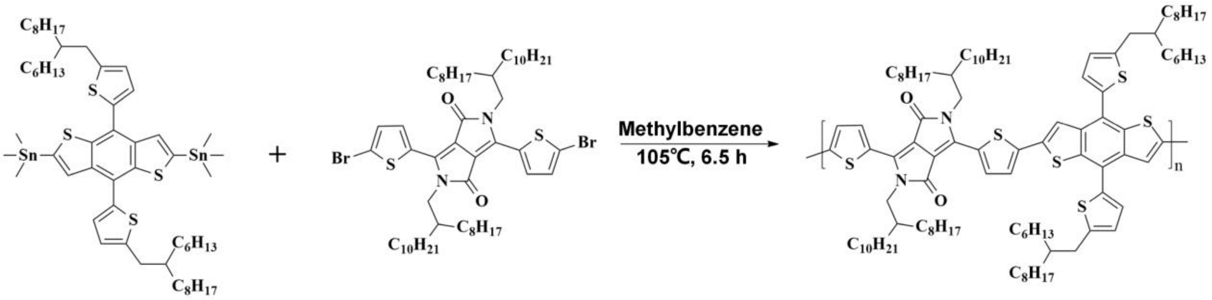

Figure S1. Synthesis route of PBDTTDPP.

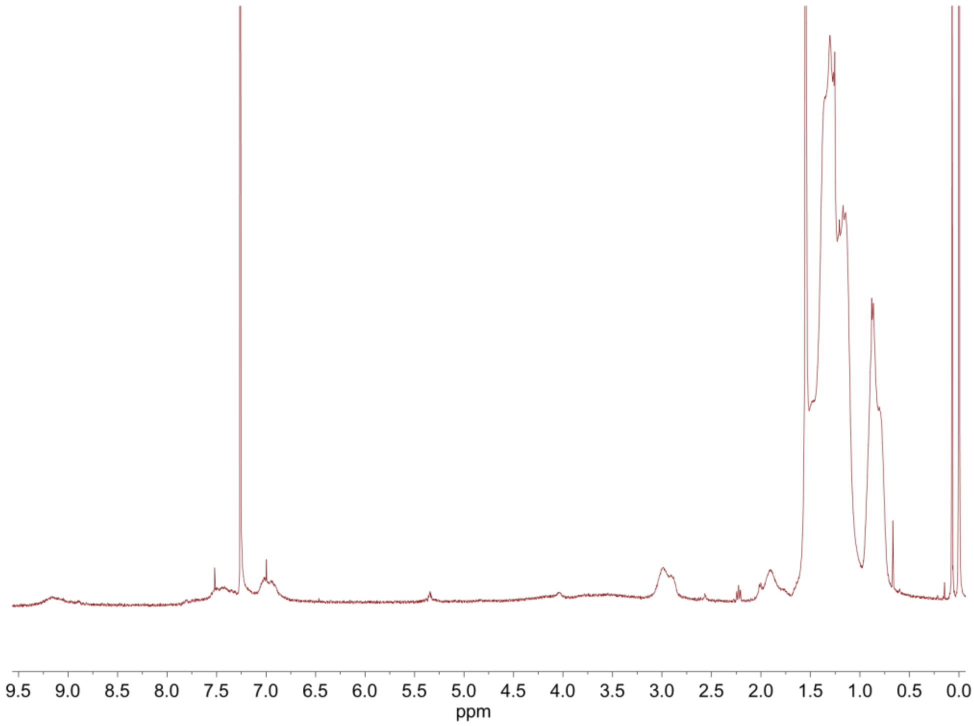

Figure S2. ^{1}H NMR spectrum of PBDTTDPP.

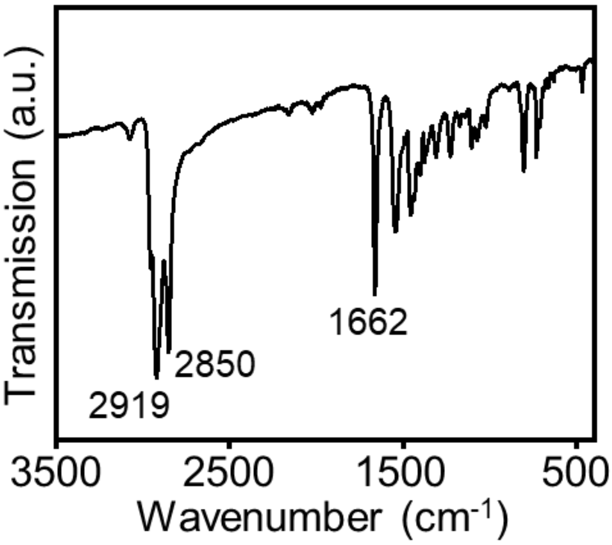

Figure S3. FTIR spectrum of PBDTTDPP. (The stretching vibrations at 2850, and 2919 cm^{-1} correspond to the C-H bonds. The stretching vibration at 1662 is attributed to the C=C bonds.)

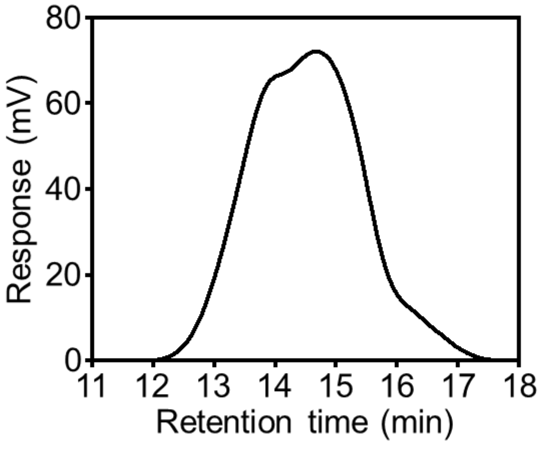

Figure S4. Gel permeation chromatography data of PBDTTDPP. Mn 25914, Mw 77418, PDI 2.9875.

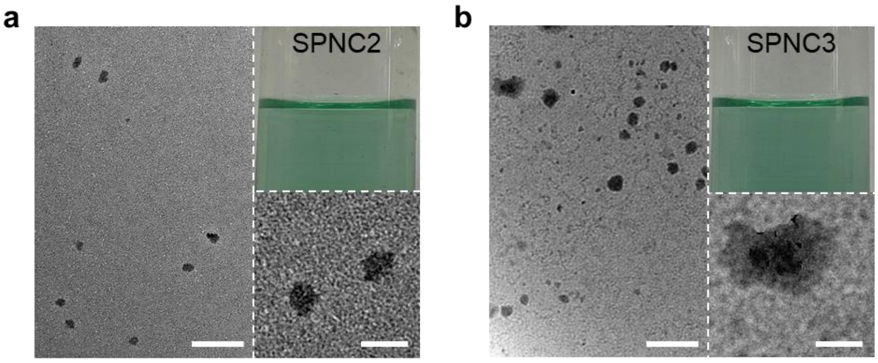

Figure S5. Nanoparticle solutions (25 μg mL^{-1}) and TEM images of SPNC2 (a) and SPNC3 (b). Scale bar (left) = 200 nm. Scale bar (right) = 50 nm.

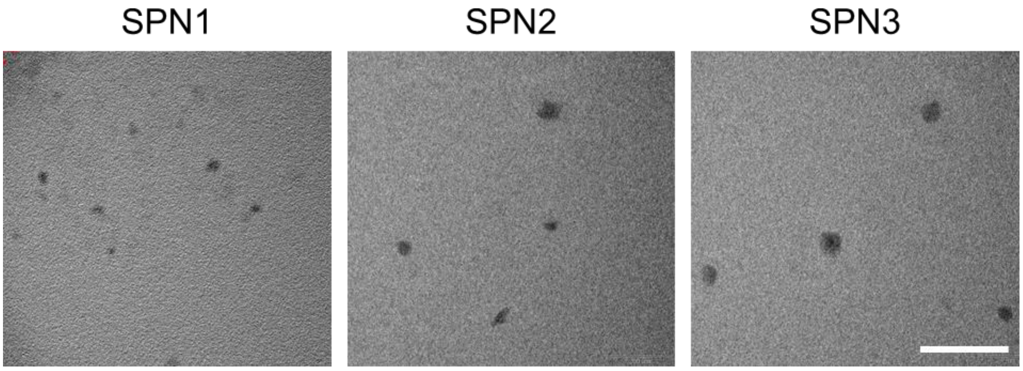

Figure S6. TEM images of SPN1, SPN2, and SPN3. Scale bar = 100 nm.

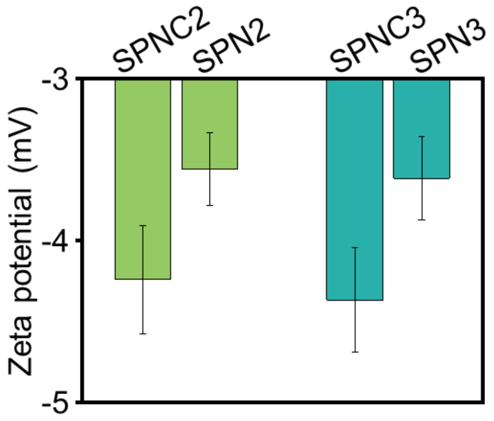

Figure S7. Zeta potential of SPNC2, SPN2, SPNC3, and SPN3, respectively. Error bars represent the standard deviations of three separate measurements (n=3).

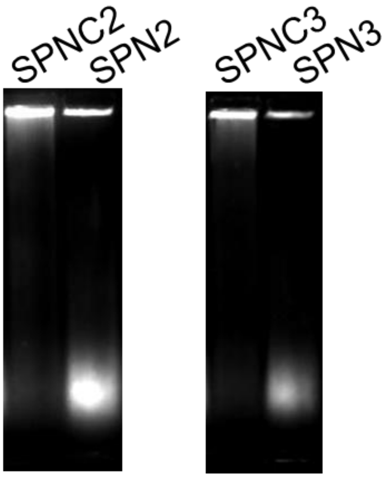

Figure S8. Agarose gel electrophoresis of SPNC2, SPN2, SPNC3, and SPN3.

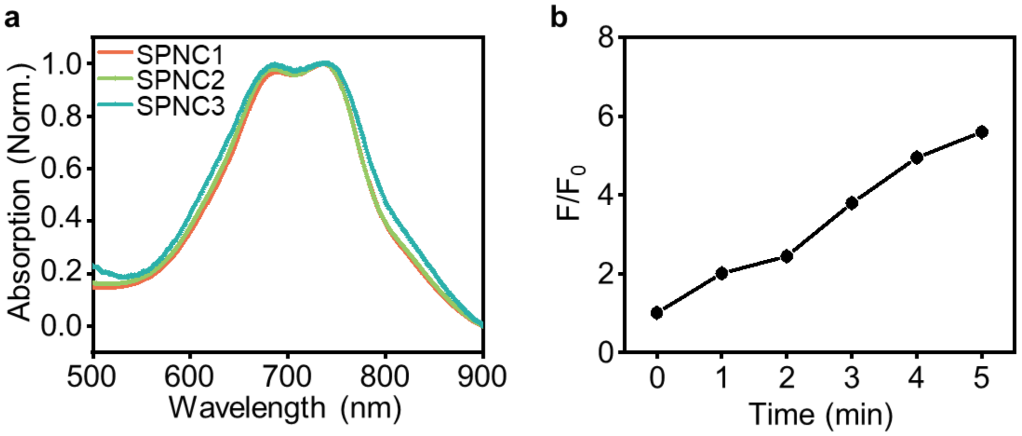

Figure S9. (a) UV-Vis spectra of SPNC1, SPNC2, and SPNC3. (b) $^1O_2$ generation of SPNC1 after different times of laser irradiation (680 nm, 0.7 W cm^{-1}) measured by fluorescence intensity enhancement (F/F_0) of SOSG.

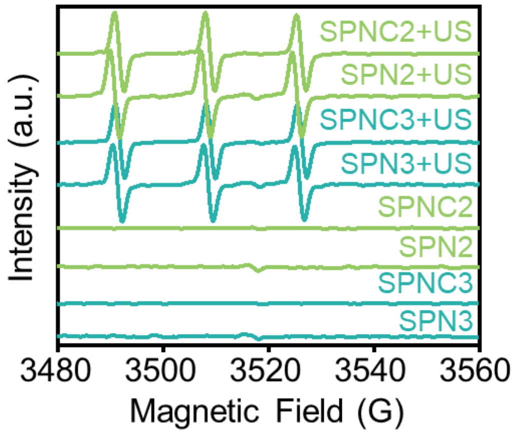

Figure S10. $^1O_2$ generation of SPN2, SPNC2, SPN3, and SPNC3 with or without US irradiation measured by ESR spectra.

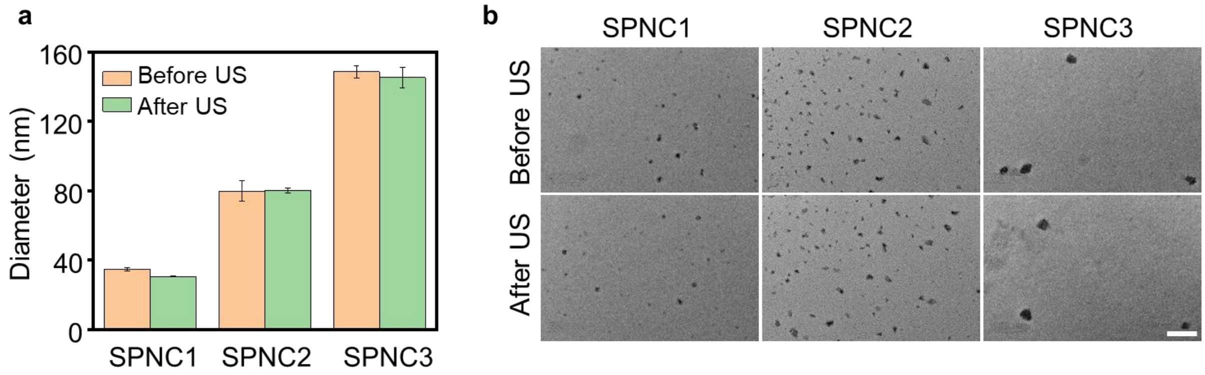

Figure S11. DLS profiles (a) and TEM images (b) of SPNC1, SPNC2, and SPNC3 before and after US irradiation for 1 min (50 kHz, 1 W cm^{-2}, 50% duty cycle). Scale bar = 200 nm. Error bars represent the standard deviations of three separate measurements (n=3).

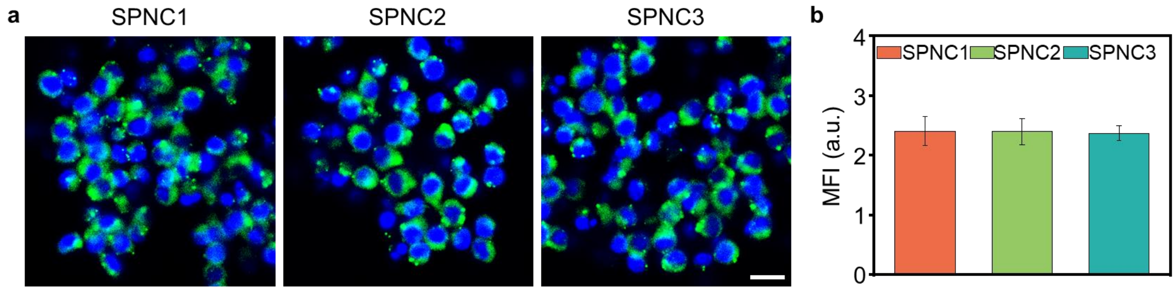

Figure S12. (a) Confocal fluorescence images and (b) mean fluorescence intensity (MFI) of macrophage cells after incubation with SPNC1, SPNC2, and SPNC3 (25 μg mL^{-1}) for 4 h. Blue fluorescence indicated the cell nucleus, and the green fluorescence indicated the signal from BODIPY doped SPNCs. Scale bar = 10 μm. Error bars represent the standard deviations of three separate measurements (n=3).

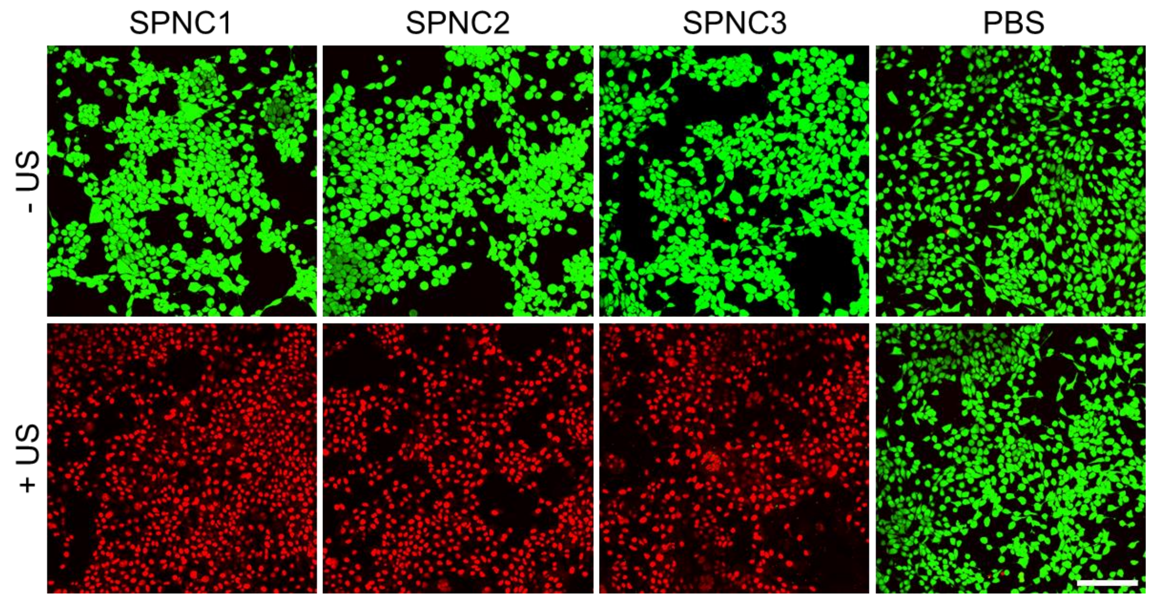

Figure S13. Confocal images of 4T1 cancer cells incubated with SPNC1, SPNC2, SPNC3 (25 μg mL^{-1}), and PBS for 4 h with or without US irradiation for 1 min. The cells were stained with calcein-AM (green fluorescence) and propidium iodide (PI) (red fluorescence). Scale bar = 100 μm.

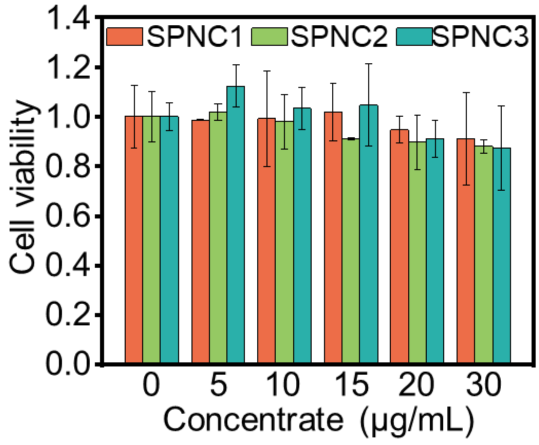

Figure S14. Cell viability of 4T1 cells after incubation with SPNC1, SPNC2, and SPNC3 at different concentrations. Error bars represent the standard deviations of three separate measurements (n=3).

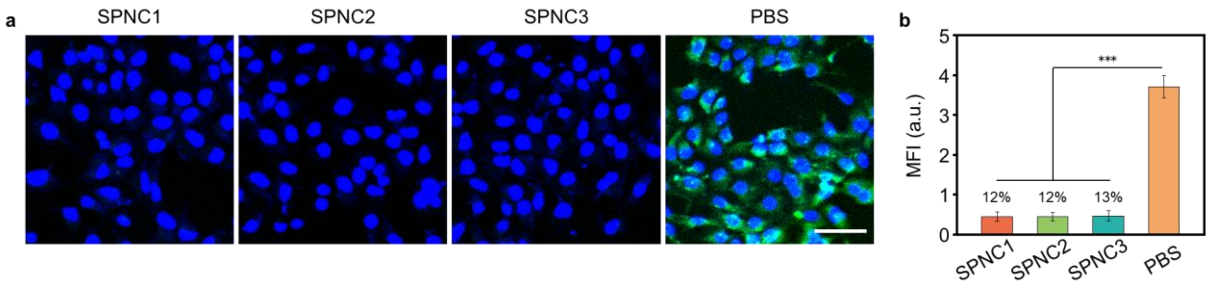

Figure S15. (a) Confocal fluorescence images and (b) MFI of 4T1 cancer cells after incubation with SPNCs (25 μg mL^{-1}), H_2O_2 (50μM), and pimonidazole for 4 h in the hypoxic bag, followed by staining with anti-pimonidazole. Blue fluorescence indicated the cell nucleus, and the green fluorescence indicated the signal from anti-pimonidazole antibody. Scale bar = 50 μm. Error bars represent the standard deviations of three separate measurements (n=3).

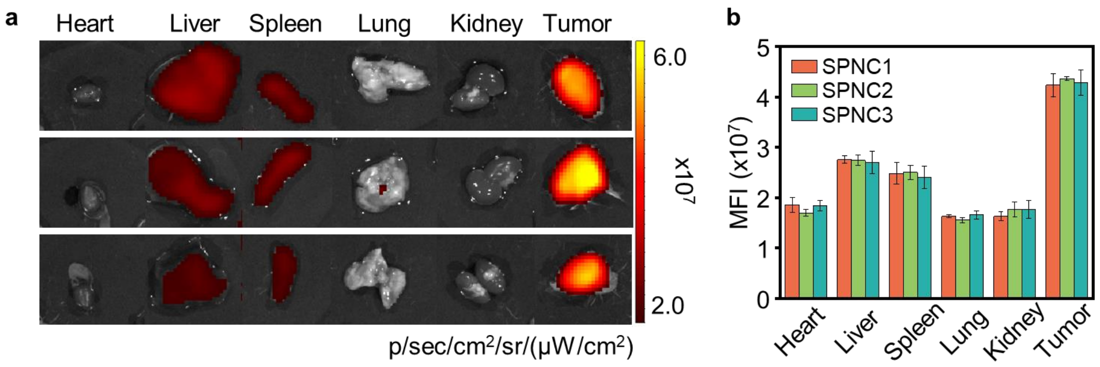

Figure S16. (a) Fluorescence images and (b) MFI of major organs of 4T1 tumor-bearing living mice after 24 h of post-injection of NCBS-doped SPNC1, SPNC2, and SPNC3 (0.2 mL, 200 μg mL^{-1}). Error bars represent the standard deviations of three separate measurements (n=3).

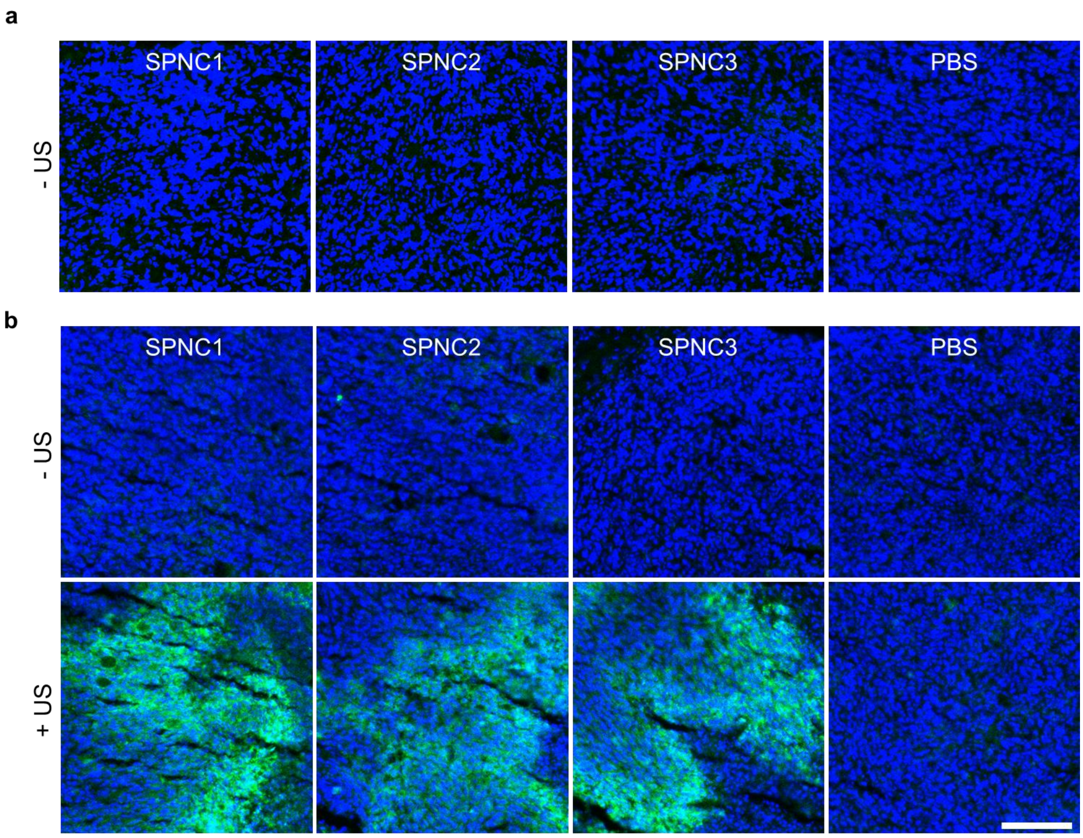

Figure S17. (a) Confocal fluorescence images of tumors from large 4T1 tumor-bearing mice after intravenous injection of SPNC1, SPNC2, SPNC3 and PBS treated with SOSG without US treatment. (b) Confocal fluorescence images of tumors from the regular 4T1 tumor-bearing mice after intravenous injection of SPNC1, SPNC2, SPNC3 and PBS treated with SOSG in the presence or absence of US irradiation. Blue fluorescence indicated the cell nucleus, and the green fluorescence indicated the signal from SOSG. Scale bar = 100 μm.

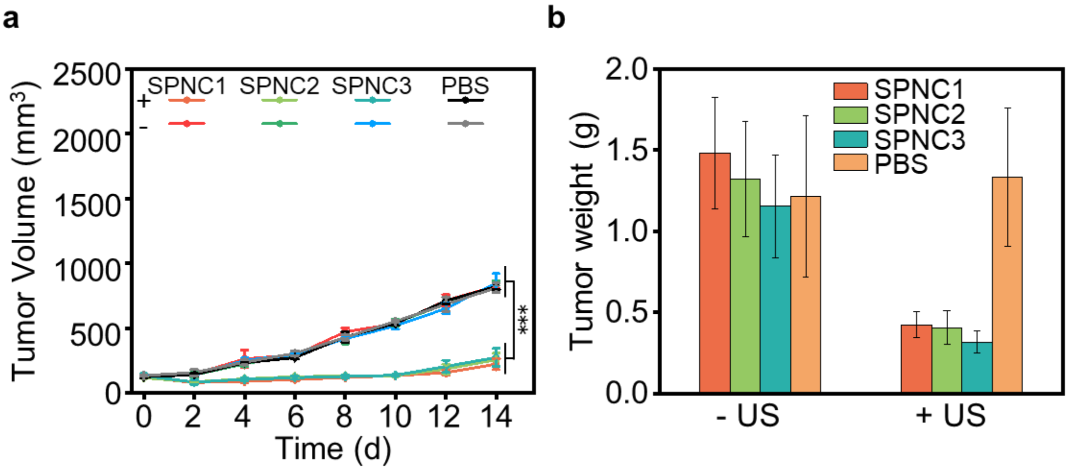

Figure S18. (a) Tumor growth curves of different groups of mice in the regular tumor model after systemic treatment with PBS, SPNC1, SPNC2, or SPNC3 in the presence or absence of

US irradiation. (b) Tumor weights of different groups of mice in the regular tumor model at day 14. Error bars represent the standard deviations of three separate measurements (n=5).

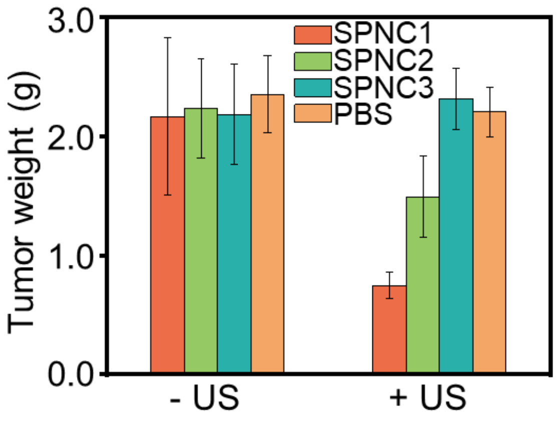

Figure S19. Tumor weights of different groups of mice in the large tumor model at day 14. Error bars represent the standard deviations of three separate measurements (n=5).

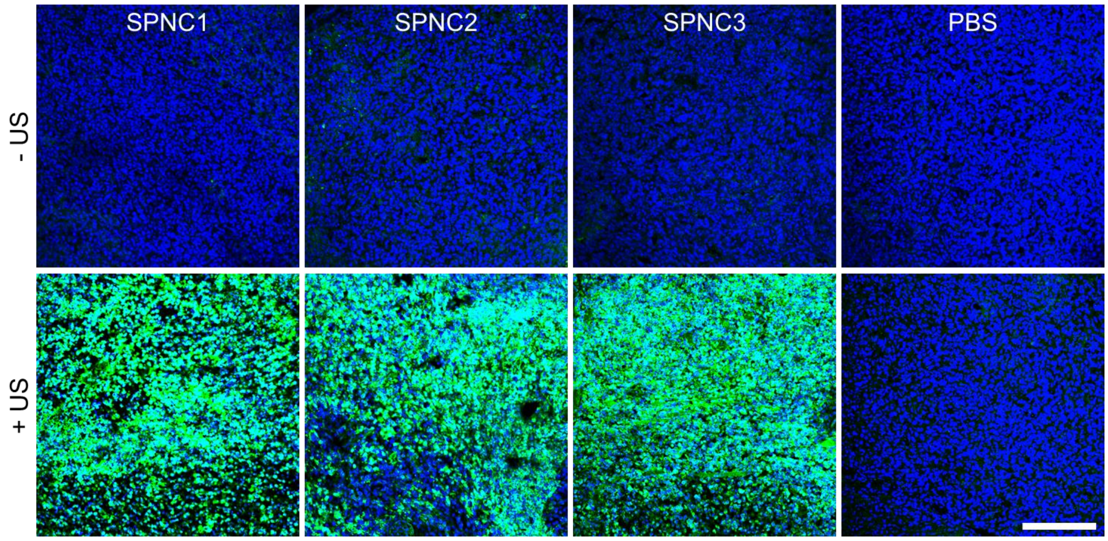

Figure S20. Immunofluorescence staining of caspase-3 of tumors from the regular 4T1 tumor-bearing mice after intravenous injection of SPNC1, SPNC2, SPNC3 and PBS in the presence or absence of US irradiation. Blue fluorescence indicated the cell nucleus, and the green fluorescence indicated the signal from anti-caspase-3 antibody. Scale bar = 100 μm.

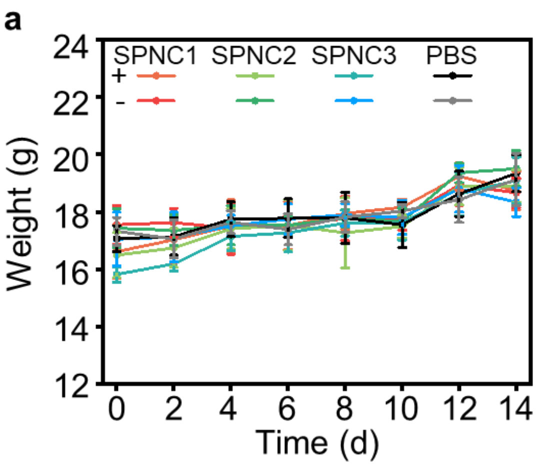

Figure S21. Bodyweight data of different groups of mice with regular(a) tumor or large tumor(b) after systemic treatment with PBS, SPNC1, SPNC2, or SPNC3 in the presence or absence of US irradiation. Error bars represent the standard deviations of three separate measurements (n=5).

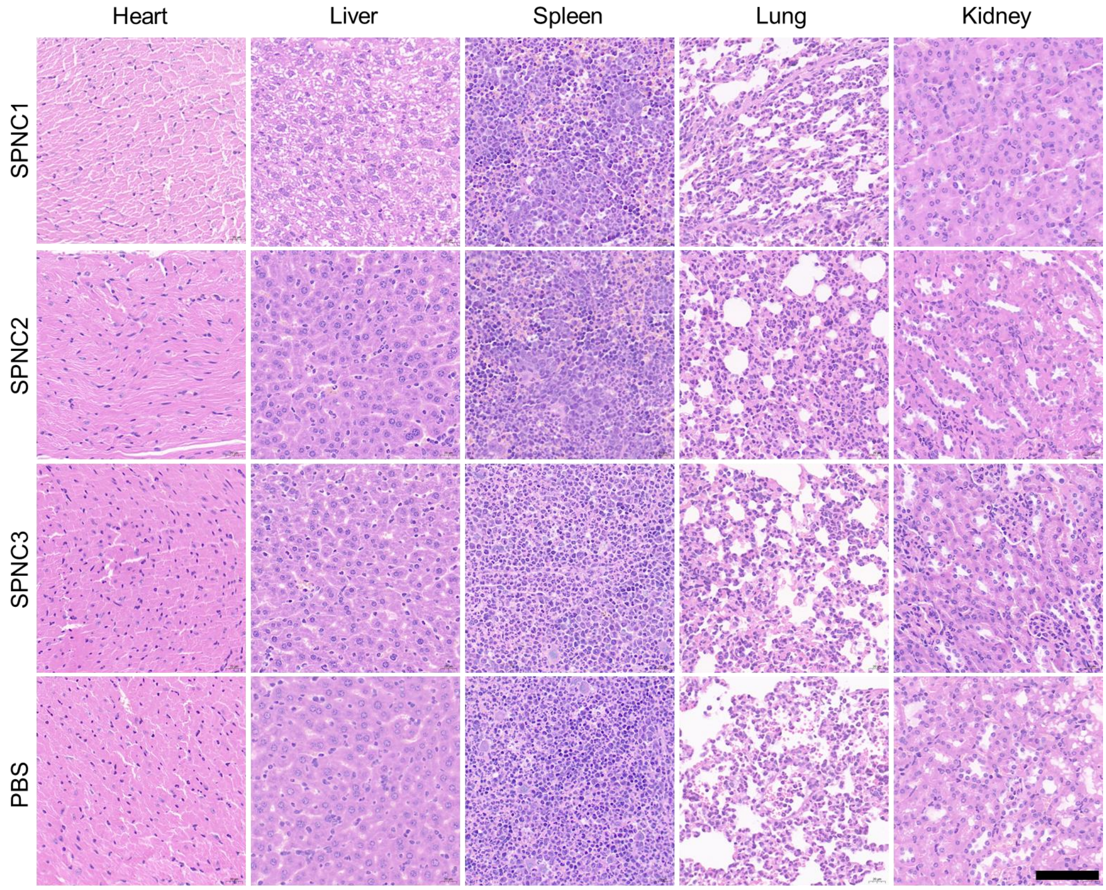

Figure S22. Histological H&E staining for major organ slices of mice at day 14 after intravenous injection of SPNC1, SPNC2, SPNC3 and PBS. Scale bar = 100 μm.

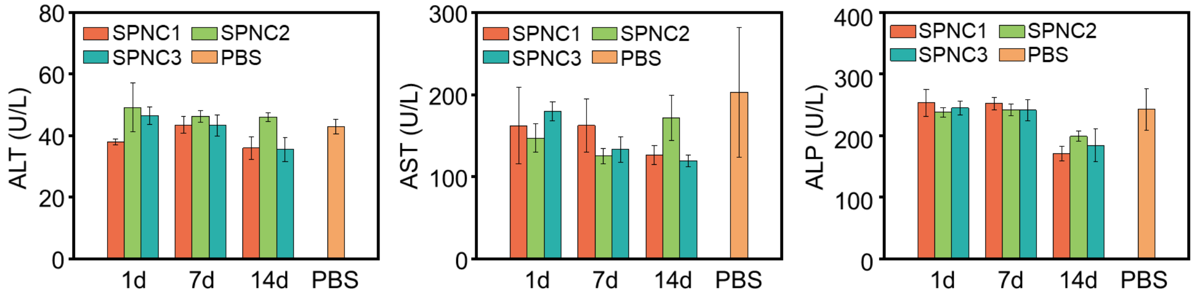

Figure S23. Biochemical analysis of liver function indicators including alanine transaminase (ALT, normal range: 10.06-96.47 U/L), aspartate aminotransferase (AST, normal range: 36.31-235.48 U/L), and alkaline phosphatase (ALP, normal range: 22.52-474.35 U/L) at day 1, day 7 and day 14 after intravenous injection of PBS and SPNCs (0.2 mL, 200 μg mL^{-1}). Error bars represent the standard deviations of three separate measurements (n=3).

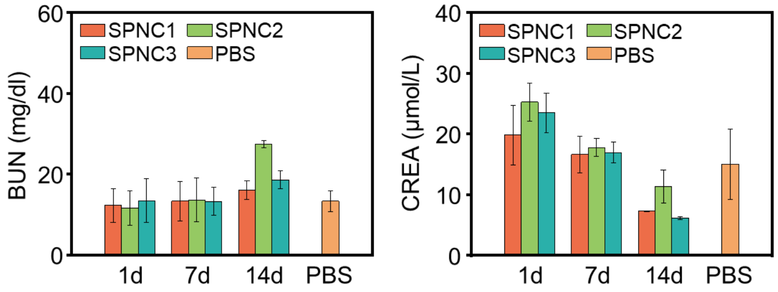

Figure S24. Biochemical analysis of kidney function indicators including blood urea nitrogen (BUN, normal range: 10.81-34.74 mg/dl) and creatinine (CREA, normal range: 5.91-85.09 μmol/L) at day 1, day 7 and day 14 after intravenous injection of PBS and SPNCs (0.2 mL, 200 μg mL^{-1}). Error bars represent the standard deviations of three separate measurements (n=3).

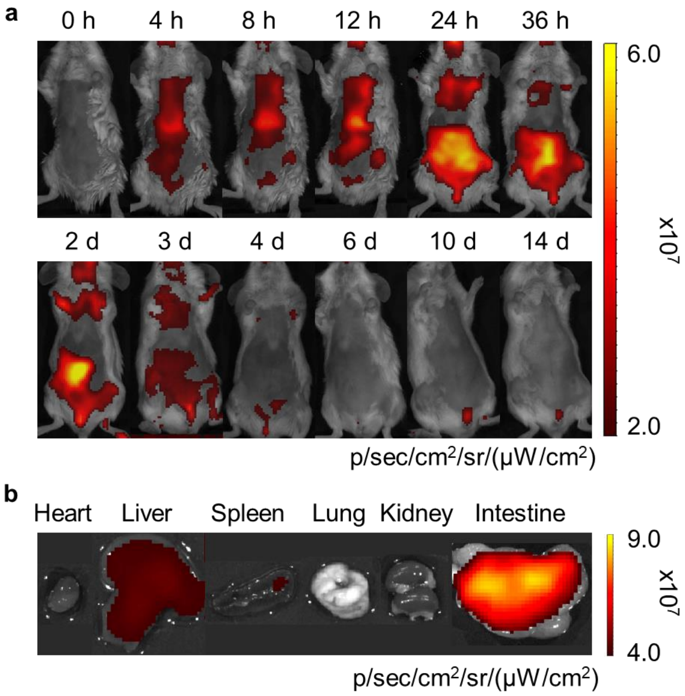

Figure S25. (a) Fluorescence images of healthy living mice at different time points after intravenous injection of NCBS-doped SPNC1 (0.2 mL, 200 μg mL^{-1}). (b) Fluorescence images of major organs of healthy living mice after 24 h of post-injection of NCBS-doped SPNC1 (0.2 mL, 200 μg mL^{-1}).

Table S1. Previous literatures that the sonosensitizers with in situ oxygen supply ability showed effective tumor growth inhibition in regular tumor models (initial tumor size: 80-120 mm^3).

Sonosensitizer materials	Initial tumor size	Oxygen improvement	Penetration ability	Tumor inhibition	Ref.
Mn-MOF	120 mm^3	√	——	√	[2]
DHMS	120 mm^3	√	——	√	[3]
GSNO/Ce6@ZIF-8@Cytomembrane	80-100 mm^3	√	——	√	[4]
LMWHA-MPB/HMME	100 mm^3	√	——	√	[5]
PpIX@HMONs-	80 mm^3	√	——	√	[6]

MnOx-RGD					
CDP@HP-T	80-100 mm^3	√	——	√	[7]
(QD@P)Rs	100 mm^3	√	——	√	[8]
SPNC1	100 mm^3	√	√	√	This work
SPNC2	100 mm^3	√	×	√	This work
SPNC3	100 mm^3	√	×	√	This work
SPNC1	360 mm^3	√	√	√	This work
SPNC2	360 mm^3	√	×	×	This work
SPNC3	360 mm^3	√	×	×	This work

3. References

[1] X. Wang, X. Zhen, J. Wang, J. Zhang, W. Wu, X. Jiang, *Biomaterials* **2013**, 34, 4667.

[2] Q. Xu, G. Zhan, Z. Zhang, T. Yong, X. Yang, L. Gan, *Theranostics* **2021**, 11, 1937.

[3] X. Pan, W. Wang, Z. Huang, S. Liu, J. Guo, F. Zhang, H. Yuan, X. Li, F. Liu, H. Liu, *Angew. Chem. Int. Ed.* **2020**, 59, 13557.

[4] J. An, Y. Hu, C. Li, X. Hou, K. Cheng, B. Zhang, R. Zhang, D. Li, S. Liu, B. Liu, D. Zhu, Y. Zhao, *Biomaterials* **2020**, 230, 119636.

[5] H. Zhang, X. Zhang, Y. Ren, F. Cao, L. Hou, Z. Zhang, *Theranostics* **2019**, 9, 3580.

[6] P. Zhu, Y. Chen, J. Shi, *ACS Nano* **2018**, 12, 3780.

[7] J. An, Y. Hu, K. Cheng, C. Li, X. Hou, G. Wang, X. Zhang, B. Liu, Y. Zhao, M. Zhang, *Biomaterials* **2020**, 234, 119761.

[8] C. Li, X. Yang, J. An, K. Cheng, X. Hou, X. Zhang, Y. Hu, B. Liu, Y. Zhao, *Theranostics* **2020**, 10, 867.
